# Figurations of Mentorship for Cultural Work

**DOI:** 10.1177/17499755251360152

**Published:** 2025-09-08

**Authors:** Frédérik Lesage, Yijia Zhang

**Affiliations:** Simon Fraser University, Canada; University of British Columbia, Canada

**Keywords:** Craft, cultural work, face-to-face, figuration, know-how, mediatization, mentorship

## Abstract

As cultural work becomes increasingly precarious and digitally mediatized, we ask how mentorship can continue to play a crucial role in shaping how the know-how of cultural work is organized and shared. Drawing on a case study of a mentorship program for cultural work taking place in Greater Vancouver, Canada, we propose conceptualizing mentorship as a figuration, which we describe by tracing three interdependent threads: a constellation of actors, frames of relevance, and communicative practice. Focusing on the constellation of actors, we analyse how the mentorship spans across a dynamic network of actors whose connected presence shape the sharing of the know-how and distributed knowledge among heterogeneous mentors and mentees. Turning to the frames of relevance, we find that mentorship is much more than a means of skill transmission. Instead, it is a site where struggles over the meaning of know-how for cultural work – frames of relevance including skill, creativity, and social justice – take place. Finally, by foregrounding the communicative practice for the figuration of this mentorship, we illustrate how mentorship is facilitated through various layers of digitally mediated exchange which reconfigure traditional notions of guidance and support for sharing experience and knowledge for cultural work. Reconceptualizing mentorship as a figuration allows us to move beyond accounts of dyadic relationships between an individual mentor and a mentee working in co-presence, and instead recognize the relational, institutional, and technological dynamics that structure contemporary cultural work. Ultimately, our findings suggest that, through the figuration of mentorship for cultural work, mentors and mentees can still share meaningful collective experiences and knowledge, which expands our understanding of the know-how necessary to practise cultural-work and how it is shared.

## Introduction

Our goal in this article is to better understand the way in which knowledge for cultural work is organized and shared as this work becomes increasingly digitally mediated. We believe that the ongoing transformation of cultural work (Ashton and Noonan, 2013; Banks, 2007; Hesmondhalgh and Baker, 2011) will crucially be shaped by if and how practitioners pass-on the ‘personal know-how’ (Dormer, 1997) they develop through experiences of working with others (Borkenhagen, 2017; Sennett, 2012). For this reason, we propose a reconceptualization of a key social arrangement for organizing how people share their know-how for cultural work: mentorship.

Mentorship traditionally involves a mentee learning know-how from a mentor by co-presently working together on shared projects. But as cultural work becomes increasingly precarious and digitally mediatized, the possibility to share the space and time needed to reproduce know-how through mentorship can no longer be taken for granted. Properly investigating the new phenomenological conditions for sharing know-how for contemporary cultural work requires that we reconsider its relational, institutional, and technological dynamics. In the following research, we demonstrate how mentorship for cultural work constitutes more than a mere means of skill transmission through shared proximity, but a figuration (Couldry and Hepp, 2016) – an interweaving of a constellation of actors, frames of relevance and communicative practice. Reframing mentorship in such a way allows us to better understand the technological mediation of know-how and the shift from face-to-face co-presence as a default social arrangement to what Couldry and Hepp term connected presence. As our findings suggest, through the figuration of mentorship for cultural work, mentors and mentees can share collective experiences and knowledge, but in ways that involve contested negotiations over what they mean and how they are technologically mediated.

In the next section, we review scholarship on craft and mentorship to conceptualize how the reproduction of know-how for cultural work has historically relied on face-to-face co-presence. We then show how this foundation is problematized by structural conditions of increasing precarity. We end the section by introducing deep mediatization as theorized by Couldry and Hepp (2016) to argue for the reconceptualization of mentorship as a figuration. In the third section, we relate findings from fieldwork for a case study of a mentorship programme taking place in Greater Vancouver, Canada, to illustrate how a figuration can be used to investigate this shift from co-presence to connected presence and to investigate its implications for how cultural workers collaborate, learn and sustain their practices. By examining the mentorship’s constellation of actors, its frames of relevance, and communicative practice, we uncover how cultural workers generate and sustain a mentor–mentee dyad to pass on know-how.

## Theorizing Mentorship for Cultural Work

### Know-how for Cultural Work

As a first step towards theorizing how mentorship is used to organize the passing on of know-how for cultural work, we turn to scholarship on craft. Definitions of craft vary wildly (Greenhalgh, 1997; Ravasi et al., 2025). The term can be used to distinguish the hand-made from the industrially manufactured (Pye, 1995 [1968]; Thompson, 1967). It is also used to distinguish amateur or folk cultural artefacts from those of professional or more artistic origins (Luckman, 2015). Scholarship on craft in the 21st century has recently hailed its reinvention (Adamson, 2019) or what Susan Luckman (2015: 18) refers to as a ‘third wave’ revival. Craft can refer to working conditions where ‘control over the conception, design and manufacture of a cultural good is possessed by individuals or small groups of workers, operating in close quarters in “workshop” conditions’ (Banks, 2007: 29), or to any ‘skilled work applied to practical ends’ (McCullough, 1996: 22) or even more simply to ‘personal know-how’ (Dormer, 1997: 139) that arises from experience of working with others. A long-standing tradition of scholarship on craft (Adamson, 2007; Baber et al., 2019; Dormer, 1997; Ingold, 2002; McCullough, 1996; Morris, 1888; Pye, 1995 [1968]; Sennett, 2008) links this know-how to a distinct attitude or set of values embodied in those who practise it that can be summed up as the patience and care needed for ‘a job done well’ (McRobbie, 2016: 131) or ‘the desire to do a job well for its own sake’ (Sennett, 2008: 9; see also Pye, 1995 [1968]: 136). It is through this attitude that craft practitioners strive for the same kind of autonomy, self-realization, and well-being that can make cultural work into ‘good work’ (Hesmondhalgh and Baker, 2011).

While not all cultural work qualifies as craft in the senses developed by the likes of Banks or McCullough cited earlier, all cultural work involves some level of craft know-how encompassing what has variously been defined as tacit knowledge (Collins, 2012; Dormer, 1997; Polanyi, 1966), situated practice (Lave and Wenger, 1991) and/or skill (Adamson, 2007; Ingold, 2002). It is no wonder then that mentorship, or what Lave and Wenger (1991) refer to as apprenticeships, as a social arrangement for sharing know-how (Chan, 2014), suits cultural work. At its core, mentorship is built around the social dyad of the mentor–mentee relationship (Dominguez and Kochan, 2020) in which the mentor passes their know-how to the mentee through a process of learning by doing (Gowlland, 2019). While the power dynamics between mentor and mentee can be more dynamic than the more traditionally familiar versions of mentorship (Dominguez and Kochan, 2020), the basic arrangement of learning by doing relies on physical co-presence. The shared space and time of this craftwork consists of a ‘task-orientation’ originally manifested in pre-industrial craft practices when ‘both work and time are intrinsic to the conduct of life itself and cannot be separated or abstracted from it’ (Ingold, 2002: 324). From this perspective, craft know-how cannot be passed on as written rules or an explicit set of prescribed procedures (Dormer, 1997; Ingold, 2013), it is only through task-oriented, co-present engagement with tools and materials that its tacit or embodied knowledge is shared.

### The Precarity of Contemporary Cultural Work

Markets, competition and technological automation have historically been presented as anathema to craft (Ingold, 2002; Sennett, 2008) and by implication anathema to mentorship (Dormer, 1997). Drawing in part on Italian autonomist scholarship on immaterial labour (Gill and Pratt, 2008), critical scholarship of the last three decades has theorized the combination of increased abstraction, flexibilization and insecurity as precarity that threatens contemporary cultural work.

While some aspects of this ‘flexible exploitation’ (Hesmondhalgh and Baker 2011: 161) have arguably always been a part of cultural work, precarity serves as a useful conceptual backdrop for understanding the digital mediation of know-how for cultural work. The spatiotemporal rhythms of task-orientation have been considered under threat long before the advent of precarity at the end of the 20th century. As early as the 19th century, industrial clock-time for the manufacture of standardized goods (Ingold, 2002; Thompson, 1967) is said to decouple the working environment from everyday life, corrupting the natural production cycles that make craft possible. Despite such transformations, some craft practices persisted or were reinvented, thanks in part through the reorganization of work and the development of new tools (Adamson, 2019; McCullough, 1996). Precarity introduces a different dominant ‘temporal profile’ (see Adkins, 2013) for cultural work which aligns to the more pragmatist-inflected ‘event’ (Thrift, 2005) or ‘project’ (Boltanski and Chiapello, 2005). Using a combination of abstracting management techniques and the affordances of information and communication technologies (Dyer-Witheford and de Peuter, 2005; Hardt and Negri, 2001; Virno, 2008), cultural work under conditions of precarity is reorganized into distributed networks of disposable labour that further undermine the sharing of know-how.

These newly prevailing spatiotemporal conditions involve a flexible time for experimentation and exploration moving towards a ‘crunch time’ (Dyer-Witheford and de Peuter, 2005) where the focus shifts to the execution of deliverables. Maintaining a traditional career becomes difficult under these flexibly exploitative conditions (Hesmondhalgh and Baker, 2011; Sennett, 2006; Taylor and Luckman, 2018), pushing cultural workers toward self-employment and putting under increasing strain their ability to develop meanings socially, politically and culturally (Gill, 2013; McRobbie, 2016). Richard Sennett (2006, 2008) argues that such insecurity undermines the possibility for practitioners to develop a sense of self through work because precarity undermines the desire to do a job well for its own sake, thereby undermining the development of what we refer to here as know-how. Furthermore, the kinds of tasks involved in cultural work also become more varied. Individuated cultural workers increasingly need to develop know-how not directly related to their craft, like finance and marketing, as a greater number of requirements and expectations are offloaded onto individuals. Meanwhile, workers seek out new sources to improve their ability to practise cultural work: from search engines to professional training, from formal to informal mentorships (Luckman and Andrew, 2020).

As presented in this section, the way in which precarity undermines the spatiotemporal conditions required for people to sustain individual careers raises questions as to whether and how these same people are able to engage in mentoring. How can experienced practitioners pass on their personal know-how to others if cultural work is organized in such a way that they cannot develop a sense of self through their work? Rather than assume the outright impossibility of the mentor–mentee dyad or assume that it can only be enacted in pre-industrial contexts or at an individual scale, we propose retheorizing the organization of mentorship by disentangling one of the key factors in the way cultural work is organized – specifically how information and communication technologies mediate, and are mediated by, culture work – which, in turn, shapes the way in which know-how is shared.

### The Deep Mediatization of Cultural Work – From Co-presence to Connected Presence

According to Paul Dormer (1997), technological mediation undermines craft know-how because it structures craft into *distributed knowledge*; ‘technology [that] has embedded within it knowledge that is not and cannot be ours to possess’ (1997: 140). Dormer attributes the origins of distributed knowledge to two factors. The first is that society is increasingly characterized by a ‘coming-together of a variety of disciplines and industries’ which spreads the knowledge required to create anything over ‘many systems of production and thought’ (Dormer, 1997: 139). The second factor is that digital tools and artefacts like application software, ‘allow us to make things without ourselves possessing the know-how to make them’ (Dormer, 1997: 139); similar to what Ingold (2002) terms the technological ‘externalisation’ of know-how and to what Kroezen et al. (2021) refer to as the ‘human–machine duality’. What this means for the organization of knowledge sharing is that: ‘In technology, knowledge is distributed especially among systems of people and hardware; in craft, knowledge is also distributed but through people alone’ (Dormer, 1997: 149).

Dormer’s perspective on technology shares many similarities with those of precarity as discussed in the preceding section. The problem with this drastic dichotomy between technology and craft, however, is that it does not leave any room for theorizing the interplay between know-how and distributed knowledge, suggesting that mentorship is impractical (if not impossible) in any highly technologically mediated society. At its most fundamental level, the dichotomy rests on an assumption that the know-how that is shared through co-presence is ‘possessed’ within the body of the mentor. And it also overlooks the possibility that distributed knowledge about work can be woven into experiential and affective dimensions for sharing know-how (Fuller, 2013).

To begin to address these issues, we turn to Couldry and Hepp’s work on ‘deep mediatization’ and figuration to reconceptualize distributed knowledge and the way contemporary mentorship enables people to share know-how for cultural work.

According to Couldry and Hepp (2016; see also Hepp, 2020) waves of mediatization from ever-increasingly complex media environments have developed into a phenomenon called ‘deep mediatization’. Deep mediatization is a manifold, ‘large “universe” of variously connected digital media through which [. . .] we actualize social relations’ (Couldry and Hepp, 2016: 34–35) that makes social domains more interconnected and multifaceted. For Couldry and Hepp, the implications of deep mediatization for work is that new kinds of working collectivities can be created with the help of digital media without the need for co-presence. Such a transformation, nevertheless, touches a long-standing concern of social theory that collectivization without community could be used to undermine or exploit workers by limiting their possibility to interact with each other (Couldry and Hepp, 2016: 168–189).

The dedicated efforts of Couldry and Hepp to articulate mediatization help us refine Dormer’s first factor for distributed knowledge. They provide a more nuanced framework for considering *how* knowledge is technologically distributed ranging from a macro to micro level:

(1) media environment: ‘the totality of communications media available at one point in space-time’ (Couldry and Hepp, 2016: 39);
(2) media ensemble: ‘the array of media that are used within a particular social domain of a collectivity or organization’ (Hepp, 2020: 89);
(3) media repertoire: ‘an individual’s selection from the media manifold as they use and appropriate media as part of their everyday practices’ (Hepp, 2020: 92).

This phenomenological ‘nesting dolls’ framework suggests that different kinds of distributed knowledge are unevenly distributed as well as more or less available to groups and/or individuals depending on the media ensembles and repertoires they can access.

Before reconceptualizing Dormer’s second factor using deep mediatization, we need to return to the dichotomy he identifies as mentioned earlier. For Dormer, the embodiment of know-how is equated with a form of tacit, human ‘possession’, whereas technologies like software mediate know-how into distributed knowledge without access to such possession. Dormer’s dichotomy relies in part on Pye’s (1995 [1968]) classic distinction between craft as a ‘workmanship of risk’, wherein the individual practitioner’s performance of tasks with tools ‘hold the key to success’ (Dormer, 1997: 138), and a ‘workmanship of certainty’, which organizes work into a technologically mediated system geared towards mass manufacture. This dichotomy encounters problems when extended to digital technologies.

Cultural work throughout the latter half of the 20th century and into the 21st has undeniably undergone a process of transformation wherein many of the activities involved in the production, circulation and appreciation of cultural artefacts are mediated by digital technologies (Lesage and Terren, 2024; Poell et al., 2021). Although many of these creative tools and platforms may be individually designed to privilege the kind of certainty that is anathema to a workmanship of risk, some have been designed to be more responsive to the needs and desires of cultural workers (Forsler and Velkova, 2018). Just as importantly, given the sheer variety of tools and platforms and the complexity of their interconnections through deep mediatization, it seems unlikely that any and all of their combinations through work qualifies as a system for producing ‘workmanship of certainty’. This complexity therefore opens up the possibility that practitioners have developed a certain kind of know-how for choosing, accessing, and using digital technologies as distributed knowledge. This requires us to re-conceptualize the social relationships that enable and constrain the interplay between know-how and distributed knowledge.

To meet this last challenge, we return to Couldry and Hepp (2016) and their claim that any enactment of relations within the complexities of deep mediatization necessarily implies a kind of ‘intermeshing’ on the part of social actors. They propose *figuration* as a means of understanding such intermeshing. Figuration has been applied in a range of sociological approaches (Haraway, 1997; Lury et al., 2022; Suchman, 2007) to investigate technological mediation. In this article, we use it to describe how practitioners engage in a complex interweaving of know-how and distributed knowledge for cultural work facing the transformations brought about by deep mediatization. When understood as a figuration, mentorship remains a dyadic relation (Hepp, 2020) between mentor and mentee, but one that is re-constituted through three facets: (1) constellations of actors, (2) frames of relevance and (3) communicative practice. Conceptualizing mentorship as figuration helps us trace how practitioners pass on knowledge within dynamically scaled media environments despite conditions of precarity. For Couldry and Hepp, figurations do not eliminate the possibility of the kinds of face-to-face, synchronous engagements required to share know-how:

The point is not that the face to face becomes less important, but that in order to sustain its primacy (for example, the importance of family meal times) we now require continuous mediated coordination, within processes of ‘connected presence’ that enable us to coordinate the possibility of that face-to-face situation. (Couldry and Hepp, 2016: 29)

Extended to the mentor–mentee dyad, the foregoing quotation implies that maintaining the primacy of face-to-face engagement as a means of sharing personal know-how for cultural work requires the development of a certain kind of ‘connected presence’, one that relies on distributed knowledge for its coordination. This connected presence would enable mentorship where ‘[e]ach individual lives at the intersection of the different figurations in which he or she is involved and develops an identity through the subjective narration of the self on the basis of his or her involvements.’ (Hepp, 2020: 102–103) As we intend to demonstrate in the following section, figuration as an analytic device is suited to investigating mentorships within conditions of precarity because it allows us to conceptualize mediated social relationships between people with heterogeneous experiences and commitments whose engagements are contingent and overlapping.

## Case Study – the MAC Mentorship

In this main section, we deploy and develop the three interconnected facets of figuration – constellation of actors, frames of relevance, communicative practice – as part of a case study of mentorship. In the next subsection, we provide an overview of the research design and methodology. In the three subsequent subsections, we define each of the three facets of figuration respectively and use them to describe the way in which know-how and distributed knowledge are shared through connected presence embedded within a media environment. Each of these sections, including the methodological overview immediately following, should be read as interconnecting facets that constitute the whole of a figuration of mentorship.

### Research Design and Methodology

The Media Arts Centre (MAC)^1^ is an artist-run centre that acts as a hub for artists and media practitioners in Greater Vancouver, Canada. It is a place where people gather in face-to-face exchanges through meetings, exhibitions and arts activities. MAC is an established institution with staff, spatial infrastructure (actual building with studios and archives) and technological infrastructure (tools, software, hardware) to support cultural work. Geared towards serving the neighbourhood in which it is located, MAC also runs a range of educational activities including a mentorship programme.

The MAC mentorship used for this case study takes place over a five-month period. It consists of multiple four-hour sessions on most Thursday evenings and Sunday afternoons, totalling 56 hours. The topic is promoted as youth-oriented podcasting focussed on the housing crisis in Greater Vancouver, designed around a collaboration with a local Urban Activism Group (UAG) whose audio archive of interviews is used as inspiration for audio projects. As a research team, we attend every regular session, unofficial meet ups of mentors and mentees, a final-touch session near the end, and a final presentation event where mentors and mentees play clips of their work for an audience of over a hundred. A member of the research team actively participates as a mentee and contributes two audio stories to the project. Apart from participant observation, a team member interviews the mentors early on, midway, and at the end of the mentorship. The team also conducts a focus group with mentees near the end, before the final-touch session. The findings presented in this article are based on a thematic analysis of fieldwork notes, interview transcripts and materials collected during the mentorship. Field notes have been coded line by line with NVivo, and thematic coding applied to transcripts to draw out facets of figuration detailed later.

### A Constellation of Actors

Consistent with traditional mentorship, the MAC mentorship is built around a human mentor–mentee dyad. However, this dyad is not embodied in two individual humans – a mentor and a mentee – but constituted through a constellation of actors. Several individuals are invited to assume the mantle of mentor by dipping into sessions to share their approach to cultural work, embodying relevant but different kinds of know-how. Similar to the mentors, mentees come to the mentorship with heterogeneous backgrounds, varying affiliation with cultural work and different levels of commitment or availability. Despite its divergence from the traditional format of mentorship, the mentorship concluded with the successful publication of mentees’ work, a well-attended community event launching the mentorship project, and mentees’ expressed enthusiasm and confidence to continue investing their commitment to cultural work despite the end of the mentorship. By zooming into the constellation of actors, the figurational approach allows a deeper dive into how know-how circulates among the constellation of actors.

The figure closest to a traditional mentor role is Gena, hired by MAC as an independent contractor to co-design the mentorship’s scope and focus as well as lead its implementation. She is physically present at nearly all face-to-face mentorship sessions to provide guidance and assistance. Gena has studied sonic arts at the graduate level, has experience in making soundscapes and podcasts, and has taught these practices to others. This is not her first time engaging in this type of freelance contract work as an artist and instructor.

Despite her expertise and experience, Gena expresses reluctance to assume herself as the sole mentor. For her, it is not enough to expose mentees only to her personal know-how, as she believes mentorship in cultural work today should not simply train mentees to replicate the mentor’s practices. Instead, she sees the MAC mentorship as a window through which mentees gain exposure to a range of people, practices and artefacts that exemplify good work, which goes beyond familiarity with a particular set of tools, software and devices. Gena’s connection with the UAG significantly shapes the format of the mentorship. Having spearheaded the creation of some of UAG’s audio archives, Gena was instrumental in securing UAG’s support to transform interviews she recorded with community residents into audio stories, the project which serves as the foundation for the MAC mentorship. Rather than a mentor who embodies know-how on their own, Gena is closer to a ‘switcher’ (Castells, 2009: 45; Couldry and Hepp, 2016: 72), connecting UAG, local residents, and audio production practitioners in her network and coordinating their contribution to the MAC mentorship.

Another ‘switcher’ in the constellation of actors is Yannis. As a MAC employee, Yannis’ job involves securing funding for mentorships, coordinating programmes, and liaising between mentors and MAC. For the MAC mentorship, Yannis occasionally participates in face-to-face sessions with mentees in the computer lab where he demonstrates audio mixing and mastering techniques. Although he embodies audio production know-how and is the go-to expert when mentees encounter challenges in producing their mentorship project, Yannis’ responsibilities go beyond this 56-hour mentorship as he oversees many other projects for the MAC. Through his involvement with other projects, Yannis accumulates connections with a wide range of cultural workers, whose unique experiences and know-how scaffold the MAC mentorship.

As ‘switchers’ in the constellation, Gena and Yannis invite guest mentors from a range of domains and practices: soundscape studies, media art, podcasting, and political organizing, which are relevant to the MAC mentorship in different ways. After the introduction of storytelling with sound, at the second session, Gena invites one of the core organizers of the UAG, whose audio archives the mentees work with, to discuss the history of the local housing justice crisis that UAG endeavours to address and the challenges to support vulnerable people in the community. When mentees begin to pitch what they would make as part of the mentorship project, Gena invites an artist with experience in audio performance, rap and poetry to lead vocal exercises and writing activities, pushing mentees to explore their responses to the entanglements between place, movement, identity and colonialism. Later in the mentorship, when mentees work with the audio materials from the UAG archive, Gena invites two audio production practitioners to handle one of the four-hour sessions on production techniques in audio journalism and another podcaster to share storytelling concepts, techniques, and his experiences producing local podcast series. Different from pedagogical designs where guest instructors disappear after delivering a one-time lecture, most guest mentors return in later sessions to provide in-person consultation support for mentees’ individual projects.

Just like the mentor in this constellation, the mentee is not embodied by a single person. Gena and Yannis put heavy weight on the diversity of mentees and how they fit into group dynamics, as they believe mentees are expected to learn from each other as much as from mentors at the project-based mentorship. Participants are considered more likely to succeed in the mentorship if they have a sense of commitment to the UAG objectives (see the next section) and a passion for cultural work. The selected mentees represent a broad age-range and have very different educational backgrounds and professional skills. Some mentees have considerable prior work experience in audio production and creative expression, while some had minimal exposure to the production side of cultural works.

What emerges from the fieldwork is that mentors and mentees are affected by conditions of precarity, but they are also able to sustain a mentor–mentee dyad through connected presence. Despite its tacitness, know-how is not ‘possessed’ by any single individual or group. Instead, know-how is shared (albeit unevenly) through moments of connected presence across the constellation.

### Frames of Relevance: Skill, Creativity and Social Justice

The second facet of figurations of mentorship consists of frame(s) of relevance for meaningfully arranging shared action; giving actors taking part in the constellation a collective sense of purpose. In a more traditional implementation of co-present mentorship, the frame of relevance would be defined by the personal know-how of the mentor as it is passed on to the mentee by co-presently working together. As we have shown in the previous subsection, the distribution of know-how across a constellation of actors makes such a frame of relevance harder to fathom. Instead, three frames of relevance, described in the following paragraphs, emerge from the fieldwork, each one consisting of overlapping yet distinct objectives and values. The first of these frames is the development of skills. The second, an engagement in a creative project. The third frame entails social justice, with a focus on the housing crisis in Vancouver. Weaving across the three frames – negotiations between mentors and mentees regarding the meaning of each frame and their relevance to the mentorship – is central to the mentorship itself. The warp and woof of entanglements between frames shift over time as priorities change and the mentees’ projects evolve.

#### First Frame of Relevance: Skill

While higher education is an important source of training for cultural work (Ashton and Noonan, 2013; Comunian and Gilmore, 2018; Salvador and Comunian, 2024), few cultural or creative professions require formal accreditation (unlike, say, engineering or medicine). There are no governing bodies that regulate the types of training available for cultural work or their quality (Ashton and Noonan, 2013). This ambiguity around accreditation helps explain why mentorships, as an opportunity to develop know-how from a more experienced practitioner, can be an attractive proposition for someone wanting to learn cultural work. But what is more difficult to capture is the ambiguity around what constitutes know-how itself. As a frame of relevance for mentorship, ‘skill’ encapsulates an empirical tension between how mentees and mentors define what know-how they need for cultural work. As we will show here, mentees look for *upskilling*, whereas mentors focus on providing *enskilment*.

Some mentees initially express insecurity and confusion about their progress through the mentorship as they expect to develop know-how with industry-specific techniques, tools, or genres. This expectation is revealed when they point to Audition, an industry-specific audio editing application software when reflecting on what skills they develop in the focus group interview close to the end of the mentorship:

Yeah Audition, definitely. I never used it before, but I was familiar with Pro Tools and Logic, but not Audition. I really like the software, actually. I think it's really intuitive for editing audio stories like this, it’s great. (Focus Group Interview)

This motivation to learn to use specific tools and techniques can be characterized as a desire to *upskill*; training to ‘professionalize’ and be ‘industry ready’. Originally conceived as a means for the working poor to get out of poverty, upskilling is rooted in a discourse on ‘new’ information, knowledge, or skills economies circulating since the 1970s (for example Bell, 1973) wherein individuals adapt their existing know-how to changing technical conditions or learn new know-how. Upskilling has since been applied to creative and cultural industries where cultural workers are encouraged to upskill to minimize the risks presented by conditions of precarity (Murray and Gollmitzer, 2012).

Gena and Yannis are uneasy with characterizing the mentorship as upskilling. They admit to continually upskilling themselves on their own time by participating in one-off training sessions, watching online tutorials, googling information, and scouring forums – something they encourage mentees to do as well (See section Communicative Practice). Both mentors also have experience providing this kind of upskilling training. But both mentors set out to provide *enskilment* for mentees in podcasting and soundscape production. Enskilment can be characterized as a non-linear development of know-how through social engagement (Gowlland, 2019; Ingold, 2002). For Gena and Yannis, this is an opportunity for mentors and mentees to explore and experiment through cultural work – in other words, to develop know-how by engaging in the ‘workmanship of risk’ – rather than to inculcate mentees with industry-specific software instruction.

When asked what skills Gena expects mentees to acquire through this mentorship, she ranks the ability to ‘listen’ and ‘storytelling’ as priorities over technical skills such as editing with Adobe Audition, using recording devices, or producing sound effects. In the following statement, she explains why mentees benefit from enskilment in listening:

[. . .] you wouldn’t go up to a bunch of tech bros and be like, ‘I know how to listen well’, right? But if you can hear what’s happening – I think like ‘tuning’ – that’s a huge skill and it’s transferable from audio editing to any kind of editing where you’re just working with understanding variations and textures and pacing and what you want and don’t want [. . .] Listening is what I’m excited about, because that’s what makes you good at doing audio: being able to listen back or hear something within a track that you can pull out and sharpen or that you want to get rid of. So, on that and storytelling and all of those things I think are first. (Gena interview)

Gena repeatedly justifies the value of enskilment over upskilling by emphasizing the former’s ‘transferability’. She perceives upskilling as overly determined by tools and techniques in a way that echoes Dormer’s critique of distributed knowledge. In this sense, upskilling can be characterized as a kind of ‘workmanship of certainty’ with distributed knowledge like application software. Conversely, enskilment like listening constitutes know-how that is harder to define, but not reliant on specific tools or techniques and thus less tied to a particular media ensemble or repertoire.

#### Second Frame of Relevance: Creativity

The ‘artist’ has long been considered the subject that maintains creative control over cultural work (Becker, 1982). As the interpolation to *be creative* (McRobbie, 2016) under conditions of precarity comes to inform the ‘ideal of the contemporary worker’ (Boltanski and Chiapello, 2005; Taylor, 2019) it seems reasonable to expect that the ideal of the romanticized (white, western, bourgeois) artist (McRobbie, 2016; Reckwitz, 2017) also shapes the meaning of mentorship for cultural work.

The MAC mentorship, however, is not about turning mentees into artists, nor is creativity considered an inherent ability possessed by the mentor that is passed on to the mentee. Instead, creative know-how is associated with gaining experience working through the *creative process*. This is a more democratic definition of creativity in that such an experience can be available to anyone. In the context of cultural work, it also constitutes a shift in meaning where creativity is no longer valued ‘as an end in itself (“art for art’s sake”) but for its practical applications, particularly in the workplace’ (Taylor, 2019: 456).

Like skill, creativity is not know-how that is the purview of a single individual, but an experience that is unevenly shared across the constellation of actors. Those with more experience are there to shepherd and encourage those with less of it. As Yannis states, the main difference between mentors and mentees with respect to engaging in the creative process as part of the mentorship is:

[. . .] that the mentees are really supposed to be the ones deciding the big creative decisions. The mentor is supporting them to make those decisions. And the mentees [. . .] in mentorships where there’s more time for people to get to know each other, they’re going to help each other too. They’re teaching each other as much as the mentor is teaching anything. (Yannis interview)

Through a connected presence with mentors and peers, mentees are supported as they experience the emotional ‘ups-and-downs’ of the creative process: the initial apprehension of not knowing where the project will lead or what steps to take at the start; the stress of pitching early ideas to others; the pressures of crunch time when finalizing the project; and, finally, the excitement of presenting the work to an audience. By engaging with others throughout the constellation of actors, mentees develop a sense of having ‘been there’, gaining the confidence to ‘power through’ these various stages.

While some mentees have experience in creative projects prior to participating in the MAC mentorship, they see this as an opportunity to expand their experiences. One mentee, for example, explains at the later stage of her project that she can now reconcile her new experiences of working with different types of audio recordings and effects with her previous experience as a writer. She acknowledges the range of ‘hard skills’ (i.e. upskilling) she developed through the mentorship, as well as a deeper comprehension of how she creates:

P1: the biggest thing I’ve taken out of it [the mentorship] is being able to learn a bit more about my creative process, how I like to work or what works for me just in a general way [. . .] I’ve definitely gained a lot of confidence with my creative work. (Focus group interview)

That mentees would have the opportunity to engage in each stage of the creative process was not a foregone conclusion. Gena’s original plan for the mentorship (shared with everyone during the first meeting) was to group mentees into teams. Each group would then develop an audio project based on interview recordings from the UAG archive. If organized this way, the project would have involved a division of labour within groups, assigning different tasks to each member – for example, some would focus on scripting, some on recording, others on editing. This subdivision would have made it easier to share the workload but, as Gena quickly realizes, it would have broken up the creative process and unevenly distributed creative control. She therefore alters the plan in week 3 so that each mentee develops their own individual project (or, in one case, a pair works together). This revised approach gives each mentee control over all stages of the project’s creative direction.

Control over the creative process, however, is relative. The MAC mentorship provides space and time for mentors and mentees to gather, pitch, prepare and discuss project stages. Gena and Yannis adapt the timeline based on mentee feedback, pushing back the deadline as projects progress. Though mentees worry about having enough time to complete their work, the mentors are less concerned with meeting specific technical or aesthetic standards typical of vocational programmes. Instead, they incite mentees to collaborate, experiment with new techniques, identify necessary materials to tell their stories, and take risks in how they tell them. The know-how being passed on is more about distinguishing what one can and cannot control, helping mentees understand when a workmanship of risk is possible (Chan, 2014; Ross and Glăveanu, 2023).

#### Third Frame of Relevance: Social Justice

Inspired in part by Raymond Williams’ cultural materialism, Hesmondhalgh and Baker (2011) argue that the factors that ensure cultural work is good work include equality, social justice and autonomy – the politics of organizing said work. The last key frame of relevance for the MAC mentorship that emerges from the fieldwork pertains to social justice, but in terms of engaging with a cause for social justice through cultural work rather than achieving social justice through its organization. To be sure, the MAC mentorship strives for equity and inclusivity in the way it is organized: how mentors and mentees are selected as well as how mentees are supported. Yet the distinction is significant because it has implications for understanding how politics, emotion and affect shape the mentorship.

As described in previous sections, commitment on the part of mentees is an important component of the mentorship. The affective register for this commitment is repeatedly framed, not as self-improvement or creativity-for-its-own-sake, but as working towards greater social justice by giving voice to issues related to the housing crisis in Greater Vancouver.

The MAC and UAG collaboration sets out to be explicitly critical of existing political economic conditions. As a central organization in the constellation, UAG and its representatives help to connect creative and political concerns that are inherently counter-hegemonic, which helps explain why there is little effort to reproduce existing industry demands. All guest mentors contribute to the social justice framing and share their insights into how this work can generate more awareness to the political cause. Gena’s own commitment to tackling housing injustice extends well before the start of the mentorship. She has worked on multiple related projects prior to taking on the mentorship including a collaboration with the UAG that served as the basis for the mentorship. As stated earlier in this article, Gena has experience running training sessions but unlike those opportunities, this mentorship gives her the chance to connect her audio know-how to her activist know-how related to a cause she holds dear:

[. . .] having some skill in audio is something I can trade for getting a lot of attention on a housing podcast [. . .] What I think – at the end of the day – the skill I think I bring to the project is really, really, really wanting to make a podcast about tenancy and then mobilising people and helping them feel excited about it and invested in it. (Gena interview)

While Gena sacrifices financial gain for creative freedom in her own practice, she does not require the same kind of commitment from mentees:

I don’t ask that for everyone, but it is cool when they bring their excitement, I would definitely say that’s appreciated. For me, I can’t speak for [Yannis], but for me it was always the [answer that applicants gave to the question] ‘Why do you want to be here?’ and reading that answer was the. . . I barely looked at the skills part of it to be honest. (Gena interview)

The extent to which mentees relate to the social justice frame is used as a barometer of their potential and ongoing commitment to the mentorship. The first face-to-face session begins with a question: ‘What is home?’. Gena asks each mentee to define or describe, in their own way, what home means for them. Mentors then link this intuitive emotional attachment to home to broader political questions of housing throughout subsequent sessions. Mentees are encouraged, even expected, to express their personal perspective on home and housing. In the second session, Gena and the mentees discuss themes emerging from the raw interviews in UAG’s archive and brainstorm additional housing-related themes based on their experiences. Mentees bring up a wide range of issues, including how single parents are an overlooked category in the existing benefits system and how some real estate developments lack access to schools. These themes serve as the basis for individual pitches that become routine among mentees throughout the sessions. Rather than distinguishing cultural work from other facets of mentee’s lives, the mentorship encourages mentees to share their personal experiences of living in Vancouver with others in the constellation and to weave this know-how into the creative process.

Many of the final projects end up only partially based on the UAG archive and its themes. Instead, mentees explore various axes of the urban housing crisis by conducting new interviews, recording fresh B-rolls (pre-recorded material that is not primary content), or using external archives. For example, one mentee interviews local seniors to collect their accounts of how the city has changed over the last few decades. Another mentee creates a soundscape using historical footage and reflects on the progress and influence of gentrification. Even in cases where mentees work with the UAG interview archive, the material is used in unexpected ways. For example, based on renoviction and ‘demoviction’ testimonials in the archive, two mentees develop a project that discusses the terminology related to various types of eviction and provides listeners with toolkits to protect housing security.

Returning to the distinction introduced at the beginning of this section, we can consider whether the emotional/affective work related to this frame of relevance constitutes the kinds of manipulative or self-exploitative affective labour described in literature on precarity and cultural work (Gill, 2013; Hesmondhalgh and Baker, 2011; McRobbie, 2016). After all, the commitment expected of actors in the figuration echo the kind of dedication expected of other cultural workers. A key difference may be the autonomy afforded to mentees in terms of exercising control over the creative process. Another significant distinction between this mentorship and, for example, the television work analysed by Hesmondhalgh and Baker is the purpose of the cultural work itself. At the risk of trivializing the cultural significance of the talent showcase they investigated, the resulting content is alienated from its creators in a way that the mentees’ work on a local housing crisis is not.

### Communicative Practice

In the two previous subsections, we established that the figuration of mentorship relies on a *constellation of actors* constituted around a mentor–mentee dyad, where each actor contributes in various ways, at various moments, to the collective *frames of relevance* that give the figuration purpose. The third facet of a figuration – *communicative practice* – refers to its ‘distinctive practices of communication and a related media ensemble’ (Couldry and Hepp, 2016: 67). The communicative practice for a ‘traditional’ mentorship, consistent with Dormer’s emphasis on the co-present transmission of know-how, involves the mentor passing on tacit know-how by engaging with the mentee in face-to-face work. Such mentorships require little technological mediation and thus little distributed knowledge. The MAC mentorship, however, uses distributed knowledge like digital media to produce connected presence, allowing mentor and mentee to share know-how while maintaining the primacy of face-to-face work as a key mode of exchange. To explore how know-how and distributed knowledge are shared differently in figuration versus traditional mentorship, we return to the nested dolls framework introduced earlier. It provides a more nuanced typology of distributed knowledge – a media environment, a media ensemble, and media repertoires – to refine the different kinds of distributed knowledge being mobilized.

The *media environment* in which the mentorship occurs is one of considerable complexity and diversity. Greater Vancouver is a relatively dense urban setting where digital connectivity and various regional and global media representations of cultural work, podcasting, creativity, and the housing crisis are common in daily life.

Within this media environment, much of the figuration’s *media ensemble* is closely tied to the MAC building, which draws together mentors and mentees from different quarters of Greater Vancouver and beyond, as well as heterogeneous technologies in one space. To access creative software tools like Adobe Audition and other pedagogical tools like computers, projector and sound systems, mentors and mentees meet at the MAC building twice a week. In the lab wired with Apple desktop computers, mentees log on a shared Google Drive to access the UAG interview archive that serves as the original source of audio materials for their creative projects as part of the mentorship, as well as worksheets, slides, and B-roll. To support mentees who want to work beyond the semiweekly sessions, Gena and Yannis allow mentees to sign out ZOOM audio recorders, provide structured training on audio production, and offer one-on-one consultation supporting mentees to produce and edit field recordings as part of their projects. The media ensemble is not limited to MAC’s physical space and hardware; support is also provided through email threads, text messages, and shared docs. The mentorship’s media ensemble also includes its representation through media artefacts like grant proposals, syllabi, and promotional materials for the final project launch. Taken as a whole, this media ensemble is closely tied to MAC as a specific organization that exists as spatial and digital infrastructure.

Consistent with Dormer’s conception of distributed knowledge, we see that the figuration’s media ensemble includes ‘people and hardware’ (Dormer, 1997: 149). However, this distribution is not organized into a system separating humans from technologically mediated knowledge but rather a complex, variegated concatenation of hardware, software, people, buildings, organizations and informational and/or aesthetic content. Instead, the media ensemble coordinates moments of connected presence in which mentors and mentees share know-how and distributed knowledge. Next, we explore two observations relating to the communicative practice of producing coordinated presence involving mentors and mentees sharing their *media repertoires* – in other words, sharing personal selections of distributed knowledge they use and appropriate as part of their work.

The first observation relates to the tension around defining skilled know-how development for cultural work as either upskilling or enskilment, discussed earlier in this article. While many technologies in the mentorship’s media ensemble, like Google Docs, social media, or emails, are equally available to mentors and mentees, key tools like Adobe Audition are only temporarily accessible on MAC computers and unavailable after the mentorship. Mentees often cannot afford subscription fees to use such software on their own devices, making it impossible to incorporate Adobe Audition into their media repertoires beyond the mentorship programme. Gena, an independent practitioner, also finds these fees prohibitively expensive. However, Gena can access the software through MAC or find alternatives due to her experience with other audio tools. This unequal access is why Gena, Yannis and other mentors focus on enskilment – sharing know-how without requiring replication of the mentor’s media repertoire. As mentioned earlier, part of the know-how exchange during connected presence involves sharing how to upskill. Mentors show mentees how to find distributed knowledge – websites, forums, YouTube channels and other resources that mentors use – to upskill independently. They encourage mentees to incorporate this distributed knowledge into their own media repertoires. In other words, although Gena and Yannis emphasize enskilment over upskilling, part of the enskilment passed on through connected presence is how to access and use distributed knowledge for ongoing upskilling. Aware of technological changes from deep mediatization, MAC mentors not only teach digital media skills (like audio editing) but also provide know-how to help mentees continually adapt to a shifting, inequitable, and complex media environment integral to cultural work.

The second observation pertains to the media repertoires Gena and Yannis use to create and maintain connected presence. Producing connected presence constitutes a kind of ‘invisible work’ (Suchman, 2002). As switchers, Gena and Yannis coordinate connected presence by emailing invitations, meeting details and reminders. They create materials on Google Docs and file repositories on Google Drive where people access and store recordings. The MAC mentors continually share and revise information through emails, face-to-face discussions and diagrams while responding to mentees’ questions and feedback. Their ability to link people, genres, skills, tools and organizations as part of the mentorship’s constellation and frames of relevance constitutes tacit know-how essential for switching and generating connected presence. Their power and influence as switchers align with networking’s growing importance as know-how for successful cultural workers (Adkins, 2013; McRobbie, 2016; Wittel, 2001). But mentors’ know-how remains ‘invisible’ to mentees because: (1) it is not performed in connected presence with mentees, and (2) Gena and Yannis’ roles as switchers are enabled partly by privileged access to MAC as a media ensemble unavailable to mentees. While some distributed knowledge for connected presence is accessible to all, it remains unclear if mentees have developed this know-how. If cultural work’s future depends on passing knowledge through mentorship, passing on know-how for switching – organizing and producing connected presence – may be critically important.

## Conclusion

Our goal in this article has been to better understand how people practice mentorship to organize and share know-how for cultural work as it becomes increasingly precarious and deeply mediatized. Given the tacit nature of craft know-how – what one must know to do ‘good work’ – mentorship remains a key way to pass on experience and knowledge. Traditionally, mentorship involved learning-by-doing through co-present work on shared projects, but this model has undergone major transformations, including increased abstraction, flexibilization, and insecurity. Disruptions to the spatiotemporal rhythms of cultural work and the erosion of its autonomy have made cultural workers more precarious. Moreover, the growing technological mediation of cultural work – what we call deep mediatization – adds complexity to co-present sharing of know-how. Scholars like Dormer argue that this complexity constitutes distributed knowledge, separate from human craft know-how. We propose that by re-conceptualizing mentorship as a figuration, it becomes more than one-on-one co-present learning. Instead, mentorship emerges as a communicative process involving a constellation of actors coordinating connected presence to share both know-how and distributed knowledge within shared frames of relevance.

While our case study (presented earlier in the article) is not universally representative, it shows how know-how and distributed knowledge are shared not only through face-to-face interactions but also through mediated, asynchronous practices.

Firstly, our findings support Couldry and Hepp’s argument that deep mediatization does not erase the importance of face-to-face contact. Shared space and time remain vital for transmitting cultural know-how – but co-presence is no longer the default. It must now be actively achieved by mentor-switchers who use communicative practices with distributed knowledge to coordinate connected presence among mentors and mentees.

Secondly, we identify three overlapping frames of relevance that give mentors and mentees a collective sense of purpose and enable coordination of connected presence. Competing interpretations of skill (upskilling and enskilment), experiencing different stages of the creative process and their effect on creative control, and generating commitment through social justice are not only frames of relevance for this figuration – they also point to broader strategies cultural workers use to mediate know-how and distributed knowledge amid precarity and deep mediatization.

Yet even in moments of connected presence, frames of relevance are neither unified nor uncontested. Mentors and mentees do not share a homogenous understanding of the skilled know-how being passed on or how it should apply to cultural work beyond the mentorship. This heterogeneity becomes especially clear in how mentors do not aim to have mentees replicate their media repertoires. Instead, Gena and Yannis focus on helping mentees learn to upskill independently by introducing them to a range of distributed knowledge. The result: mentors and mentees could share know-how without sharing the same media repertoires.

These findings challenge a traditional assumption of mentorship – that its value lies in mentors and mentees developing a shared phenomenological experience of work through close contact. Our exploration of mentorship as a figuration, describing the phenomenological intermeshing through which people share know-how and distributed knowledge amid deep mediatization, shows that despite complexity and inequity in media ensembles and repertoires, mentors and mentees can still share experiences and knowledge with the support of organizations like MAC and the constellation of actors it connects.

## Notes

1. The names of organizations and individuals have been changed to ensure confidentiality for all research participants.
